# Low-Calorie Ketogenic Diet: Potential Application in the Treatment of Polycystic Ovary Syndrome in Adolescents

**DOI:** 10.3390/nu15163582

**Published:** 2023-08-15

**Authors:** Valeria Calcaterra, Hellas Cena, Francesca Sottotetti, Chiara Hruby, Nagaia Madini, Noemi Zelaschi, Gianvincenzo Zuccotti

**Affiliations:** 1 Department of Internal Medicine and Therapeutics, University of Pavia, 27100 Pavia, Italy; valeria.calcaterra@unipv.it; 2 Pediatric Department, Buzzi Children’s Hospital, 20154 Milano, Italy; chiara.hruby@unimi.it (C.H.); gianvincenzo.zuccotti@unimi.it (G.Z.); 3 Laboratory of Dietetics and Clinical Nutrition, Department of Public Health, Experimental and Forensic Medicine, University of Pavia, 27100 Pavia, Italy; francesca.sottotetti@unipv.it (F.S.); nagaia.madini@unipv.it (N.M.); noemi.zelaschi01@universitadipavia.it (N.Z.); 4 Clinical Nutrition and Dietetics Service, Unit of Internal Medicine and Endocrinology, ICS Maugeri IRCCS, 27100 Pavia, Italy; 5 Department of Biomedical and Clinical Science, University of Milano, 20157 Milano, Italy

**Keywords:** polycystic ovary syndrome, diet, nutritional intervention, ketogenic diet, low-calorie ketogenic diet, very-low-calorie ketogenic diet

## Abstract

Polycystic ovary syndrome (PCOS) is the most common endocrine disorder in women of reproductive age. Hyperandrogenism, hyperinsulinism and insulin resistance (IR) are the main drivers of clinical, metabolic and reproductive phenotypes of PCOS. In adolescence, the cornerstones of PCOS treatment are lifestyle and dietary interventions. In particular, the quality and quantity of carbohydrates introduced with the diet play a crucial role in the benefits of diet on PCOS. Recently, the ketogenic diet (KD) has attracted significant interest for the treatment of IR and for the control of carbohydrate metabolism, which has proven to be beneficial for several dysmetabolic conditions, including PCOS. The goal of the KD is to induce a fasting-like metabolism with production of chetonic bodies. Ketosis is a good regulator of calorie intake and mimics the starvation effect in the body, leading to body weight control and consequent metabolism. Additionally, during ketogenesis, insulin receptor sensitivity is also promoted. We proposed a broad overview of the available literature regarding KD indications and considered its metabolic benefits useful for improving PCOS management. The reported data support that a low-calorie ketogenic diet (LCKD) plays a positive role as a regulator of control weight, IR, glucose and lipid homeostasis and hormonal profile. Unfortunately, the evidence concerning the benefits of the very LCKD in adolescents with PCOS and excessive body weight is still numerically scarce. Further studies are necessary to understand whether these effects are due to weight loss or to the nutritional characteristics of this diet. Considering the long-term consequences of PCOS, it is crucial to detect the prospects of nutritional interventions to protect fertility, starting in adolescence.

## 1. Introduction

Polycystic ovary syndrome (PCOS) is the most common endocrine disorder in women of reproductive age [1,2]. The epidemiology of PCOS is still unclear; diagnosed cases involve up to 13% of women of reproductive age, but variability in clinical presentation and diagnostic criteria lead to great underdiagnosis, which is believed to involve 70% of affected women [2]. The disorder is associated with significant reproductive, metabolic and psychological consequences.

The pathogenesis of PCOS remains not fully elucidated, and it is thought to be led by both exogenous and endogenous factors, with hyperandrogenism, hyperinsulinism and insulin resistance (IR) being the main drivers of clinical, metabolic and reproductive phenotypes [3]. IR and hyperandrogenemia can establish a vicious cycle with each stimulating the other, providing the basis for the metabolic therapy of these patients [ref].

Limited data are available regarding the diagnosis, assessment, treatment and prognosis of PCOS in the adolescent population, although early identification of the disease’s hallmarks may bring substantial benefit in terms of cardiovascular prognosis and fertility [4].

Treatment options for PCOS differ upon targeted manifestations, according to the patient’s phenotype and priorities (i.e., fertility, hyperandrogenism, obesity). In adolescence, cornerstones of PCOS treatment are weight loss through lifestyle and dietary interventions, followed by pharmacological or surgical therapy [4].

Concerning dietary intervention, the key point of benefits is the effect of diet composition on insulin sensitivity. Dietary carbohydrate intake influences postprandial glucose levels and consequently the insulin [5]; additionally, the anti-inflammatory properties of diet are relevant to endocrine and metabolic profiles [6]. It is important to highlight that any diet that guarantees weight loss can improve IR, hyperandrogenism and fertility in PCOS women. The obesity-induced metabolic impact of PCOS phenotype appears to be reversible after weight loss [7].

The quality and quantity of carbohydrates introduced with the diet play a crucial role in the benefits of diet on PCOS. The Mediterranean diet (MD) is a dietary pattern rich in complex carbohydrates and fiber, and high in monounsaturated fat, that is closely associated with an improvement in low-grade chronic inflammation, IR, and hormonal derangements in PCOS [4].

Recently, the ketogenic diet (KD) has attracted significant interest in the treatment of IR and for the control of carbohydrate metabolism. The KD is an isocaloric, high-fat and hypoglycemic diet that has been increasingly adopted in recent years for specific pathologic conditions, such as refractory epilepsy. The goal of KD is inducing a fasting-like metabolism with production of chetonic bodies which has proven to be beneficial for several dysmetabolic conditions, including type 2 diabetes mellitus, cardiovascular disease, and ultimately PCOS [7]. Ketosis is a good regulator of calorie intake and mimics the starvation effect in the body, leading to body weight control and additional consequent benefits which affect insulin resistance related to obesity [8]. During ketogenesis, insulin receptor sensitivity is also promoted; therefore, KD can modulate insulin fluctuation and secretion which are also caused by decreased carbohydrate intake, ultimately leading to improved insulin sensitivity [8,9,10]. In addition to the positive regulation of glucose metabolism, KD may also result in enhanced lipolysis and fatty-acid oxidation, leading to a better lipidic profile [8].

We proposed a broad overview of the available literature regarding low-calorie KD indications and its metabolic benefits on PCOS management, focusing on the adolescent population. Since PCOS is one of the leading causes of anovulatory infertility in women, it is important to pay attention to the childbearing age. A review of the data on prospects of nutritional interventions in PCOS care is important for fertility protection, starting in adolescence.

## 2. Methods

We performed a narrative review, presenting a non-systematic summation and analysis of the available literature on the topic of the application of low-calorie KDs in women with PCOS, focusing on adolescents [11]. The most relevant original scientific papers, clinical trials, meta-analyses and reviews published in the last 15 years were considered. We also imposed restrictions of the English language and the human species. The exclusion criteria used were the following: in vitro studies, animal studies, elderly populations, epilepsy, and malnutrition by default. For this purpose, we carried out a search for relevant papers on three databases (MEDLINE/PubMed, Scopus and Web of Science) using a combination of text and MeSH (medical subject headings) to enhance search strategies using specific keywords, such as polycystic ovary syndrome, adolescents, diet, nutritional intervention, ketogenic diet, low-calorie ketogenic diet, and very-low-calorie ketogenic diet.

Starting from a total of 201 papers, three authors assessed the titles and abstracts (n = 69) and consequently reviewed the full texts of relevant studies (n = 41). We resolved any disagreements by consultation with a fourth author. The reference list of all manuscripts was also considered to identify relevant manuscripts.

## 3. Polycystic Ovary Syndrome in Adolescents

PCOS presents a high prevalence in adolescents as in adult women, despite the high incidence of infertility in the affected population. Reports on the epidemiology of PCOS in adolescents are rare, and evaluation of its prevalence in adolescents may be difficult because of the high prevalence of paraphysiologic ovulatory dysfunction and of the common echographic finding of micropolycystic ovaries in this population; prevalence data depend on which diagnostic criteria are used and on the observed population [12]. A recent systematic review and meta-analysis that involved almost 150,000 adolescent girls around the globe stated that the prevalence of PCOS may be around 11% according to the Rotterdam criteria, with a higher prevalence in Middle-Eastern and South American women and lower prevalence in Asian populations [13].

Growing evidence suggests that adult diagnostic criteria are not useful in PCOS assessment in the first 8 years after menarche; application of the Rotterdam criteria may easily result in overdiagnosis, and radiological and biochemical assessments in adolescent girls may be challenging due to the lack of data about androgen reference levels and normal ovarian morphology in this age range. Moreover, echographic evaluation in young women is limited to the transabdominal approach; acne also shows generally high prevalence in adolescence, whereas hirsutism is less frequent than in adult women.

Widely accepted criteria for diagnosing PCOS in the adolescent population are still lacking, but early recognition and intervention may bring significant benefits in terms of reducing the incidence of non-communicable diseases such as obesity and diabetes mellitus, other than reducing cardiovascular risk and increasing fertility [14].

An Australian study published in June 2023 aimed to redefine reference values for the Rotterdam diagnostic criteria in adolescents (up to 8 years after menarche); the population consisted of 226 post-menarcheal girls (median age 15 yrs) whose clinical, echographic and biochemical data were collected [15]. The authors defined cut-offs for free androgen index, free testosterone, menstruation length and modified Ferriman–Gallwey score for clinical signs of hyperandrogenism. The population was subsequently divided into two clusters: cluster 1 was characterized by higher levels of androgens and clinical hyperandrogenism, which correlated to higher BMI than cluster 2, and was diagnosed with PCOS. It is important to underline that cut-off values were lower than those applied to adult populations, and that the clinical hyperandrogenism score was lower in PCOS adolescents than in adults, according to what has been previously reported in the literature regarding the longer time needed for the pilosebaceous glands to respond to high androgen levels after puberty [15].

Pathogenetic mechanisms of PCOS in adolescence are complex and result from both genetic predisposition and environmental factors, leading over the years to the definition of the “two hits model”.

Although the role of genetics is thought to be limited and a pattern of inheritance has not yet been identified, familiar aggregation has been widely reported. It has been estimated that 25% of adolescents with PCOS have an affected mother, and several studies reported a higher incidence of PCOS in monozygotic twins than in dizygotic twins or siblings exposed to the same environment [16]. Multiple genes have been linked to an increased risk of PCOS, mostly involved in ovarian steroidogenesis, follicular maturation and the insulin-signaling pathway. A recent family-based study of genome-wide association conducted on an Italian population (n = 212) was able to find several variants associated with PCOS; candidate genes were involved in PCOS phenotype traits, such as obesity, insulin resistance, impaired ovulatory function, gonadotropin level regulation and infertility [17]. Other than PCOS predisposition itself, polycystic ovarian morphology is generally inherited in an autosomal dominant fashion [18].

Epigenetic mechanisms have also been proposed as potential parts of the pathogenetic process; several animal models have shown higher incidence of PCOS following antenatal (as well as early postnatal period) androgen exposure [19]. Animal models confirm that in utero exposure to androgen excess may result in metabolic conditioning to PCOS-like phenotype in offspring, and a recent study from Risal et al. [20] demonstrated a five-fold increase in risk of developing PCOS in mice born from mothers with PCOS. Although further studies are needed to assess the effect on humans, higher serum levels of dehydroepiandrosterone (DHEAS) and testosterone in the first years after menarche have been found in daughters of PCOS women than in controls in several studies [21,22].

Moreover, pregnancy in PCOS women presents a higher incidence of complications such as aberrant placentation, leading to chronic hypoxia and higher risk of gestational diabetes mellitus and intrauterine growth retardation (IUGR), which negatively impact on the fetus’s metabolic programming with increased risk of IR and cardiovascular morbidity later in life [20].

A three-step pathogenetic pathway has been proposed that suggests an adaptive role of PCOS: the first step appears to be connected with low birthweight and early excessive postnatal weight gain with percentiles crossing, which causes adipocyte proliferation and fat tissue deposition in between the viscera (central adiposity). The second step is thought to occur in later childhood, when adipocytes secrete mediators that induce early growth and maturation as a compensating mechanism for excessive nutrients, with possible adrenarche and hypothalamus–pituitary axis activation up to precocious puberty. The third step occurs during adolescence (postmenarchal phase) and is characterized by physiological growth deceleration, with persistence of adipocyte mediators and classical PCOS appearance. Due to this pathogenetic hypothesis, a new acronym was recently proposed: “Postpubertal Central Adiposity Syndrome” [23]. It has also been hypothesized that this adaptive mechanism may have conferred an evolutionary advantage in ancient times, allowing for earlier beginning of fertile age [24].

IR and subsequent compensatory hyperinsulinemia is also thought to play a central role in PCOS reproductive and metabolic manifestations, with a prevalence of 70–80% in overweight women and of 30% in lean women [25]. Noticeably, IR in PCOS does not involve ovarian tissue, leading to an increase in its gonadotropic effect: insulin acts as a co-gonadotropin through an indirect mechanism, enhancing luteinizing hormone (LH) action on theca cells, and through a direct mechanism that induces higher hypothalamic secretion of LH, leading to increased dehydroepiandrosterone (DHEA) and androstenedione production [26].

Obesity is seen in around 50% of PCOS patients, and significant differences have been identified according to ethnicity [27]. Although the higher prevalence of IR in overweight women suggests a BMI-dependent pathogenesis, obesity and overweight have also an independent role in disease phenotype and metabolic disruption. In women with obesity, the disordered folliculogenesis of PCOS results from hyperandrogenism; hyperinsulinemia with IR. It is difficult to completely distinguish insulin resistance from hyperandrogenism in PCOS as they usually accompany each other [28]. In fact, IR and hyperinsulinemia in PCOS patients may result in hyperandrogenism via several pathways [28], including promotion of androgen synthesis from theca cells, decrease in hepatic production of SHBG, interference with GnRH signaling or adrenal androgen production; on the other hand, evidence for the converse situation, i.e., androgens adversely influencing insulin action, has been also described [29]. Additionally, obesity is known to trigger low-grade inflammation, which in turn enhances IR. Furthermore, leptin secretion by adipocytes causes ovarian aromatase inhibition, with lower androgen-to-estrogen conversion rate, and also has a negative effect on folliculogenesis [3]. Finally, gut microbiota alterations have been widely described in obesity, and several studies have described decreased abundance and variability in both lean and overweight PCOS patients, suggesting a possible new therapeutic frontier [30].

Other important contributors to PCOS pathogenesis are hormone-like substances of exogenous source, such as endocrine-disrupting chemicals (EDCs), which are almost ubiquitous in plastic recipients and packaging. Antenatal exposure to these substances has been strongly associated in recent decades with higher risk of low birth weight, preterm birth, genitalia malformation, childhood obesity and reproductive tract diseases, including PCOS [31,32,33].

PCOS is often associated with psychosocial problems such as anxiety disorder, depression and eating disorders, particularly binge-eating and bulimia nervosa; some authors have hypothesized that PCOS may be induced by epigenetic mechanisms triggered by dietary imbalance in early adolescence [34]. Unbalanced diet may also affect the gut microbiota’s composition, contributing to the previously mentioned pathogenetic mechanisms [35].

Currently, no specific pharmacotherapy has been recommended from either the Food and Drug Administration (FDA) or the European Medicines Agency (EMA) for the treatment of PCOS features in adolescents. Historically, in adults the most prescribed agents are oral contraceptives, but in recent years scientific research has been focusing on different agents, such as metformin and anti-androgenic drugs.

Metformin is widely used off-label in patients with metabolic impairments, given its insulin-sensitizing activity through inhibition of hepatic gluconeogenesis and increase in peripheral tissues’ glucose uptake [36,37]. Despite favorable clinical effects both on hyperandrogenism features and menstrual cycle regularity, metformin showed no superiority when compared to lifestyle intervention alone or combined oral contraceptives [38,39].

Antiandrogens have been widely studied because of the high psychosocial impact of androgenic features such as acne and hirsutism in adolescence. Most prescribed agents act through blockading androgen receptors (i.e., spironolactone) or through 5a reductase inhibition (i.e., finasteride). Spironolactone showed greater efficacy than metformin in menstrual cycle regulation, while studies on finasteride in the adolescent population are still lacking [40].

A recent systematic review assessed the efficacy of different pharmacological interventions on several PCOS features: no statistically significant difference in menstrual regularity was noticed between the interventions, while combination therapies led to more significant reduction in HA symptoms [38]. A meta-analysis by Wu et al. compared the metabolic effect of oral contraceptives and combination of oral contraceptives plus metformin in non-obese adult PCOS patients, and concluded that the combination of the two drugs achieved significant reduction of hyperinsulinemia, although the effect was mostly due to hepatic insulin clearance and not to a real reduction in insulin sensitivity, which showed no difference between the two groups [41].

In adolescence, first-line interventions remain lifestyle improvements such as physical exercise, dietary intervention and, only when necessary, pharmacotherapy.

Physical activity (PA) can bring significant benefits for metabolic disruption in PCOS women by reducing fat tissue and cardiovascular risk and increasing insulin sensitivity, other than important collateral benefits on psychosocial health and self-perception. The most popular and beneficial type of PA is aerobic activity (both continuous and intermittent) at a submaximal heart rate, for at least 75–150 min in normal-weight women depending on exercise intensity, and 150–250 min in overweight/obese patients, for an average of 16 weeks. PA showed efficacy in reducing weight, insulin resistance, hyperandrogenism features and increasing fertility in adult women [42].

The diet represents a crucial intervention for PCOS management and treatment. A 2020 meta-analysis concluded how important it is to provide professional dietary advice to all PCOS patients since diet is able to reduce IR and improve body composition [43].

The diet cornerstone consists of a low-calorie diet, with the objective of weight loss in overweight patients and weight maintenance in lean ones. In this regard, the American Association of Clinical Endocrinologists and American College of Endocrinology Guidelines [44] recommend a weight loss of 5–10% or more in women with PCOS in order to positively affect ovulation, regulate menstrual cycles, reduce hirsutism, improve IR, and decrease androgen levels. However, specific data on weight loss in adolescents are still not available.

The low-glycemic-index diet has also [44] shown a positive impact on inflammatory marker decreases and IR improvement, as also confirmed by others [45,46]. It is important to focus not so much on the total intake of carbohydrates, but more on the type of carbohydrates that are consumed, since a diet rich in unrefined foods and soluble fiber has been associated with greater insulin sensitivity [4]. It has long been established that the Mediterranean diet (MD) has important anti-inflammatory and antioxidant properties because of the adequate intake of fibers, unsaturated fats and vitamins and bioactive compounds [47]. Polyphenols have shown efficacy in reducing inflammatory response and insulin secretion in PCOS [48,49].

In patients with PCOS, the benefits of anti-inflammatory and antioxidant properties of omega-3 fatty acids, such as α-lipoic acid and N-acetylcysteine, on insulin sensitivity were also described [50]. Additionally, the role of dietary micronutrients, such as minerals and vitamins, on IR modulation could be also considered [4].

However, more recently, other dietary interventions have been explored, such as the ketogenic diet, which will be discussed in the next section.

## 4. Ketogenic Diet

The KD was drafted for the first time in 1920 as a dietary approach to the treatment of patients with drug-resistant epilepsies or with epilepsies secondary to rare metabolic diseases, such as GLUT-1 deficiency [51].

Over the years, globalization has led to general economic growth, greater access to food and an increase in physical inactivity; the main consequence of this process is the increased prevalence of non-communicable diseases (NCDs), including obesity, metabolic and cardiovascular diseases and cancer. In this regard, the ketogenic diet has been used for the treatment of the aforementioned pathologies, showing positive impact [52].

The ketogenic dietary approach may be tailored considering different “protocols”, including the classic ketogenic diet (CKD), the low-calorie ketogenic diet (LCKD), the very-low-calorie ketogenic diet (VLCKD), the isocaloric ketogenic diet (ICKD) and the modified ketogenic diet (MKD) [51].

KD composition is based on a reduced intake of carbohydrates (30–50 g/day), with different ratios between fat and protein intakes [51] aiming to induce ketosis, a condition in which the body is compelled to use ketone bodies (synthesized from fat) as an energy substrate instead of glucose [53]. When the KD is also low in energy, rapid weight loss and improvement of the metabolic profile have also been identified, mainly due to decreased insulin levels and increased glucagon levels, in addition to ketosis [54].

Furthermore, KD modifies the metabolic pathways of the subject, reducing lipogenesis and increasing lipolysis, influencing adipose tissue deposition and lipid plasma levels.

The KD has also been associated with antioxidant effects, generating a reduced amount of ROS (reactive oxygen species) and reshaping gut microbiota [6].

Regarding patients affected by obesity, the very-low-calorie ketogenic diet (VLCKD) has been associated with a significant reduction in body weight and BMI, waist circumference and fat mass [55].

The rapid weight loss could be due to the impact of ketone bodies on appetite with decreased perception of hunger and, consequently, in a higher adherence to smaller food portions and less food consumption overall. In fact, KD acts on the central nervous system, regulating eating behavior [6].

Furthermore, the KD has been shown to lead to the reduction of hyperinsulinemia and the improvement of insulin sensitivity in patients with type 2 diabetes [56]; the improvement of patients suffering from non-alcoholic fatty liver disease (NAFLD) is instead determined by the reduction of insulin resistance due to the increased use of fatty acids in the ketogenesis process with consequent reduction of the synthesis of intrahepatic triglycerides (IHTG) [57].

Since several KDs have been developed over time, it is difficult to provide a standard definition of a “ketogenic protocol”. In this regard, Frigerio F. et al. [51] presented different types of KDs and classified them according to their composition, focusing primarily on energy and lipid intake. Others classify KDs according to the desired ketogenic ratio (KR), which is calculated in grams of fat to grams of protein plus carbohydrates, ranging usually from 1:1 to 4:1 [6,51].

In particular, regarding the VLCKD, it is divided into different phases involving the exclusion of many foods, so following this diet with normal food products could become monotonous and ineffective. In this regard, many companies are specializing in the production of foods modified from a technological point of view which, despite being low in carbohydrates, allow the patient’s choices to be broadened, thus making them more compliant with the proposed scheme. These products include soups, smoothies and bars, but also ready meals with controlled portions; these foods are therefore essential in controlling the daily caloric intake [58].

In the first phase, patients follow a VLCKD (around 600 and 800 kcal/day) or a LCKD (>800 kcal/day and <total energy expenditure) (50); the latter is preferred to meet needs and avoid deficiencies in adolescents with PCOS [56,59].

This phase usually lasts from 8 to 12 weeks, depending on the individual and the agreed weight loss [56].

The next phase consists of a gradual reintroduction diet in which previously excluded foods are reintroduced into the patient’s diet, starting with foods with the lowest glycemic index (fruit, dairy products), followed by foods with a moderate glycemic index (legumes) and high-glycemic-index foods (bread, pasta and cereals) [2].

Fundamental in this phase is the figure of the professional (doctor, dietician...), who accompanies the patient on a path of nutritional education in order to implement a balanced diet. This has a twofold objective: the continuation of slimming and the maintenance of the weight achieved, and the promotion of a healthy lifestyle to improve the individual’s well-being and prevent the onset of NCDs [55].

As already underlined, KD provides for the exclusion of foods, including cereals and derivatives, fruit, various types of vegetables and some dairy products. It follows a reduced intake of some vitamins and minerals, such as calcium, vitamin D, potassium, sodium and magnesium, for which patients could develop deficiency. Because of this, it is essential to be integrated with a specific vitamin/mineral supplementation [52].

In order to avoid these consequences, the followed parameters also need to be monitored before, during and at the end of the diet:

– Physical examination (anthropometric measurements, blood pressure, heart rate, etc.);
– Laboratory analysis (complete blood count, creatinine, uric acid, glucose, lipid profile, sodium, potassium, calcium, magnesium, inorganic phosphate, thyroid stimulating hormone (TSH), free- thyroxine (FT4), 25-Hydroxyvitamin D, complete urinalysis and microalbuminuria) [52].

Although transient, there are several side effects that can appear in subjects who follow a KD. Indeed, the reduction in energy and carbohydrate intake and the consequent formation of ketone bodies can lead to the onset of fatigue, headache, hypoglycemia and halitosis. The lack of fiber intake can instead lead to constipation and intestinal discomfort. However, these effects usually resolve within a few days to weeks of KD initiation [55,56].

## 5. Ketogenic Diet and Polycystic Ovary Syndrome

Among the interventions for the treatment of PCOS that have been shown to have a positive impact on cardiometabolic health, decrease in androgen levels and menstrual irregularity, the modification of lifestyle is of particular importance, including physical activity, diet, regular sleep and weight loss when needed [4].

In patients with excessive weight, achievable goals, such as 5% to 10% weight loss within six months, led to significant clinical improvements [2].

For example, Marzouk et al. demonstrated that dietary weight loss in adolescent women with a body mass index (BMI) greater than 30 kg/m^2^ led to significant improvement in menstrual regularity, hirsutism score and anthropometric parameters [60].

An improvement in the metabolic profile and a decrease in circulating testosterone was observed also by Gower et al. after a modest reduction in dietary carbohydrate intake [61,62].

In a 2013 study, Mehrabani et al. showed that both a balanced hypocaloric diet and a high-protein, low-glycemic-load, hypocaloric diet led to significant weight loss and reduction in androgen levels, and the second type of diet led also to improvement in insulin sensitivity [62,63].

In a 2020 meta-analysis evaluating whether low-calorie diets improved insulin sensitivity in women with PCOS, a greater reduction in insulin resistance (IR) was reported when a low-carbohydrate diet was prescribed rather than a high-carbohydrate diet [64].

This could be explained by the fact that excessive carbohydrate (CHO) intake leads to constant low-grade inflammation associated with IR and hyperandrogenism, resulting in increased production of reactive oxygen species (ROS), oxidative stress and inflammation [65].

This is also supported by the randomized controlled clinical trial conducted by Mei et al. in 2022 on a sample of 72 overweight women with PCOS (aged between 16 and 45), who followed a MD combined with a low-carbohydrate (LC) dietary model for 12 weeks, observing an improvement in anthropometric and endocrine parameters [65,66].

Therefore, among various dietetic patterns, ones with low CHO intake such as different KD protocols have been considered by some authors in women affected by overweight or obesity and diagnosed with PCOS.

**Figure 1:**
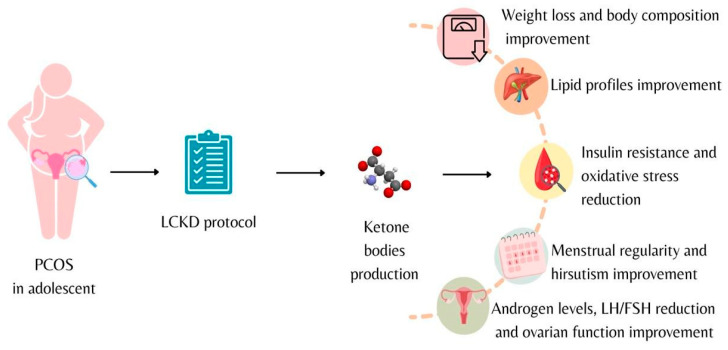
Low-calorie ketogenic diet effects on adolescents diagnosed with polycystic ovary syndrome.

In a 2013 review, Paoli et al. suggested how KD may have therapeutic action in some metabolic and endocrine diseases, including PCOS, by improving hyperinsulinemia and associated consequences, such as hyperandrogenism [6,66,67]. The potential effects of KD are summarized in Figure 1.

One of the first studies regarding this topic is a pilot study conducted in 2005 on a sample of 11 women of childbearing age (18–45 years old), diagnosed with PCOS with mean BMI 38.5 kg/m^2^. This study evaluated the effects of a LCKD followed for 24 weeks, showing that in the five women who completed the study, there was a significant reduction in body weight, percent free testosterone, LH/FSH ratio, fasting serum insulin and symptoms. Furthermore, improvement of ovulatory function led to pregnancy of two women after 4 weeks [67,68].

In the last few years, studies conducted in 2020 by Paoli et al. [68], in 2021 by Cincione et al. [69] and in 2022 by Magagnini et al. [70] have shown that the use of different protocols (in terms of duration and composition) of KD in a population aged, respectively, 16–45, >18 and 18–45 of overweight and obese women diagnosed with PCOS led to statistically significant results on endocrine and metabolic parameters.

In particular, in all the considered studies, after treatment with the KD for 12 weeks and 45 days, improvements were demonstrated in anthropometric parameters and body composition [69,70,71].

A significant reduction of the HOMA index was observed as well as in lipid profile (total cholesterol, low-density-lipoprotein cholesterol, triglyceride levels), along with an increase in high-density-lipoprotein cholesterol (HDL-col).

Interesting results have also been shown in hormonal and ovulatory function, with significant increase in sex-hormone-binding globulin (SHBG) and significant reduction of circulating androgens such as free testosterone and dehydroepiandrosterone (DHEAs).

In all three studies, an increase in estradiol and progesterone, a reduction in the luteal hormone/follicle-stimulating hormone (LH/FSH) ratio and a significant reduction in the anti-Müllerian hormone (AMH) were also found [69,70,71].

These outcomes were confirmed by a 2023 randomized controlled trial, lasting 16 weeks, aimed at evaluating the efficacy of a very-low-calorie ketogenic diet (VLCKD) compared to a balanced low-calorie diet (LCD) in women of childbearing potential (18–45 years) with PCOS suffering from obesity [72].

In conclusion, studies regarding the use of VLCKD in women with PCOS and excessive body weight are still numerically limited, but they are rapidly increasing.

In Table 1, the main studies on effects of KD on PCOS included in the narrative review are resumed.

However, considering the studies discussed in this paper, the VLCKD in this target population has showed a positive impact on multiple short-term clinical aspects (weight and fat mass loss, IR reduction, improvement in glucose, lipid homeostasis, reduction in androgen levels and LH/FSH ratio and increase of SHBG).

This was also reported in a 2019 systematic review of the “Italian Society of Endocrinology” [52], which, however, concludes that the use of the VLCKD in patients with PCOS affected by obesity presents only preliminary evidence, and therefore the degree of recommendation appears to be limited.

Despite this, there are still some concerns about the potential risks of the VLCKD, and the studies conducted so far regarding its use in the treatment of obesity, also in children and adolescent populations, show some limitations, including: protocols studied in the short term and high dropout due to possible side effects; poor compliance due to the strict dietary pattern and its application in daily life (lack of acceptance of certain foods, poor palatability, ...); economic impact and consequences on the social sphere; risk of onset of eating disorders (ED) from the VLCKD (as a very selective dietary pattern).

In this regard, a less drastic dietary model in terms of energy intake, such as a LCKD, may be necessary in order to avoid the possible undesirable effects mentioned above and any nutritional deficiencies, which could affect the correct development of this target population.

Nonetheless, the potential effects that the state of ketosis determines on the decrease in appetite and the increase in the sense of satiety should not be underestimated: this condition could decrease the number of dropouts and determine a positive response to the clinical–nutritional outcomes [52,55].

The “Italian Society of Endocrinology”, in 2019, stated that such a dietary protocol should only be suggested to patients with PCOS and excessive body weight who do not respond to a low-energy-balance diet in order to improve IR, ovulatory dysfunctions and hyperandrogenism [52].

Furthermore, in this population, the evaluation (baseline, intermediate and final) of the levels of important micronutrients (zinc, magnesium, selenium, vitamin D, B12, chromium and folic acid) should be considered in order to avoid deficiencies and possible negative consequences [4].

## 6. Conclusions

The reported data support that the KD plays a positive role as a regulator of control weight, IR, glucose and lipid homeostasis and hormonal profile. Unfortunately, the evidence concerning the benefits of VLCKD in adolescents with PCOS and excessive body weight is still numerically scarce. Further studies are necessary to understand whether these effects are due to weight loss or to the nutritional characteristics of this diet [42]. Considering the long-term consequences of PCOS, it is crucial to detect the prospects of nutritional interventions that can help with personalized preventive strategies to protect fertility starting in adolescence.

Given the beneficial effects found in the adult population and the lack of clinical trials evaluating the effect of the VLCKD diet in adolescents with PCOS, it could be useful to implement studies in this age group which may alternate VLCKD cycles with periods of balanced hypocaloric diet and permanent lifestyle modifications, evaluating long-term outcomes.

## Figures and Tables

**Table 1:** Main studies on effects of ketogenic diet on polycystic ovary syndrome included in the narrative review.

First Author, Year of Publication	Title	Study Type	Population	Intervention	Results
Paoli et al., 2020 [61]	Effects of a ketogenic diet in overweight women with polycystic ovary syndrome.	Single-arm study (interventional)	24 overweight women with PCOS Age: 18–45 yo	Participants followed a ketogenic diet (KD) for 12 weeks	KD as a possible therapeutic aid in PCOS
Cincione et al., 2021 [62]	Effects of Mixed of a Ketogenic Diet in Overweight and Obese Women with Polycystic Ovary Syndrome.	Interventional study	17 overweight and obese women with PCOS Age: 18–45 yo	Participants were treated for 45 days with a modified KD protocol, defined as “mixed ketogenic”	KD improves the anthropometric and many biochemical parameters (LH, FSH, SHBG, insulin sensitivity and HOMA index) and reduces androgenic production
Magagnini et al., 2022 [63]	Does the Ketogenic Diet Improve the Quality of Ovarian Function in Obese Women?	Retrospective study	25 women with PCOS and first-degree obesity Age: ≥18 yo	Participants followed a VLCKD protocol for 12 weeks	Metabolic and ovulatory improvement is achieved in a relatively short time
Pandurevic et al., 2023 [64]	Efficacy of very-low-calorie ketogenic diet with the Pronokal^®^ method in obese women with polycystic ovary syndrome: a 16-week randomized controlled trial	Randomized controlled trial	32 childbearing age women with PCOS, BMI 28–40 kg/m^2^ Age: 18–45 yo	Experimental group (n = 15): VLCKD for 8 weeks then LCD for 8 weeks, according to the Pronokal^®^ method; Control group (n = 15): Mediterranean LCD for 16 weeks	In obese PCOS patients, 16 weeks of VLCKD protocol with the Pronokal^®^ method was more effective than Mediterranean LCD in reducing total and visceral fat, and in ameliorating hyperandrogenism and ovulatory dysfunction

## Data Availability

Not applicable.
